# Silicone Resin Polymer Used in Preventive Maintenance of Asphalt Mixture Based on Fog Seal

**DOI:** 10.3390/polym11111814

**Published:** 2019-11-05

**Authors:** Peide Cui, Shaopeng Wu, Haiqin Xu, Yang Lv

**Affiliations:** State Key Laboratory of Silicate Materials for Architectures, Wuhan University of Technology, Wuhan 430070, China; cuipeide@whut.edu.cn (P.C.); xuhaiqin@whut.edu.cn (H.X.); lvyang@whut.edu.cn (Y.L.)

**Keywords:** silicone resin, fog seal, 3D reconstruction, moisture susceptibility, high-temperature stability

## Abstract

The commonly used materials in fog seal are emulsified asphalt and modified emulsified asphalt. Nevertheless, there are some intractable problems including aging under ultraviolet, poor permeability, and moisture susceptibility. Therefore, silicone resin polymer was used as a kind of innovative fog seal material in this study. Physicochemical properties of solidified silicone resin were characterized. X-ray computed tomography and 3D reconstruction technology were used to evaluate permeability and distribution of silicone resin polymer in an asphalt mixture. Moisture sensitivity and high-temperature performance of the asphalt mixture maintained by silicone resin polymer were also detected. The results show that surface characteristic of silicone resin can effectively isolate moisture, thereby improving moisture resistance of the asphalt mixture. Silicone resin was found to be evenly distributed throughout the pores of a sample when the dosage was 400 or 600 mL/m^2^. The pore filling rate increased by 16.3% when the dosage was changed from 200 to 400 mL/m^2^, whereas it only increased by 3.7% when dosage increased from 400 to 600 mL/m^2^. Moisture damage resistance of asphalt mixtures generally increased as the dosage of silicone was increased. However, as the dosage increased from 400 to 600 mL/m^2^, the growth rate in residual Marshall stability (RMS) and tensile strength ratio (TSR) slowed significantly since the pore filling effect of silicone has reached the upper limit. Dosage of silicone resin has little effect on the results of the rutting test while it has significant influence on Hamburg wheel tracking test (HWT). Furthermore, it was found that 400 mL/m^2^ is the optimum silicone dosage for open-graded friction course (OGFC)-13 mixture in consideration of permeability, distribution, performance of mixture, and economic cost.

## 1. Introduction

Highways based on asphalt mixture are the main type of highways in the world due to their flatness, low noise, and driving comfort [1,2,3]. However, various distresses will appear with the extension of service time under the influence of traffic load and environment [4,5,6,7]. By the end of 2018, the maintenance mileages were 4,475,800 km in China, accounting for 98.2% of total mileages of highway, indicating that the maintenance task is extremely heavy. Preventive maintenance refers to active maintenance operations before the occurrence of distress for maintaining road performance and postponing major rehabilitation. Compared with traditional passive corrective maintenance, it can not only allow traffic to resume quickly, but also save more than 45% of maintenance costs [8,9,10,11].

There are many kinds of preventive maintenance technologies, including fog seal, slurry seal, micro-surfacing, thin overlay, and ultra-thin wearing course [12,13,14,15]. Among them, fog seal is the most convenient technology to construct and fastest to resume traffic [16,17]. Its maintenance principle is to infiltrate fog seal material into the interconnected voids and close them, heal the small cracks, and prevent moisture from corroding the asphalt mixture. Therefore, the key to fog seal technology is the development of fog seal material. Currently, emulsified asphalt and modified emulsified asphalt are commonly used materials in fog seal. In addition, some rejuvenescent components are added to regenerate asphalt on pavement surface and improve the low temperature crack resistance. Jeong Hyuk and Y. Richard [18] compared curing time and adhesive behavior of polymer-modified emulsions (PMEs) with unmodified emulsions. It was found that PMEs showed higher emulsion curing rates and better aggregate retention than unmodified material. Nadeem A. et al. [19] investigated the effect of fog seal on the performance of open-graded friction course (OGFC) and the results show that abrasion resistance of OGFC pavement was improved by fog seal while surface friction was reduced. Meanwhile, $0.10\;\mathrm{gallon}\cdot {\mathrm{yard}}^{-2}$ is the optimal dosage to enhance rutting and moisture resistance according to the Hamburg test. Readul Mohammad et al. [20] evaluated the reduction in hydraulic conductivity and skid resistance after fog seal treatment of emulsions. The results show that fog seal has a significant potential to reduce hydraulic conductivity and causes an obvious drop in skid resistance.

Nevertheless, there are some intractable problems of using emulsified asphalt in fog seal, including aging of emulsion under ultraviolet, poor permeability, and moisture susceptibility [21,22,23]. Therefore, it is of great significance to explore innovative fog seal materials. Silicone resin polymer has been widely used in the field of waterproofing due to its extreme hydrophobicity [24,25,26]. It is a kind of polymer material with Si–O–Si bonds as a basic structure and at least one organic group is directly connected to silicon atoms. The unique structure of the silicone resin combines the properties of organic and inorganic materials such as excellent moisture resistance and permeability. It can penetrate the interior of the asphalt mixture and solidify to provide a maintenance effect. Therefore, silicone resin can feasibly be used as a fog seal material. Lin et al. [27] investigated the functional group of silicone resin and its effect on moisture sensitivity of the asphalt mixture. The results indicated that the Si–O is the main functional group in resin and silicone material could increase the retained Marshall stability and tensile strength. Zhu et al. [28] used liquid silicone resin to treat the recycled aggregate made of demolition waste. The results indicated that the pretreatment of recycled aggregate by silicone resin could improve the strength, absorption, and surface morphology of recycled aggregate.

At the present time, the physicochemical properties of the silicone resin used as a fog seal material have not been fully explored, and permeability and distribution law in mixture are not known. Meanwhile, the effect and mechanism of silicone materials on asphalt pavement maintenance are not clear. Based on the above background, objectives of this study are to:

1. Characterize the physicochemical properties, including surface micromorphology, element composition and distribution, and physical phase of solidified silicone resin polymer by means of SEM (scanning electron microscopy), XRF (X-ray fluorescence), EPMA (electronic probe microanalyzer) and XRD (X-ray diffractometer).
2. Investigate the permeability and distribution of silicone resin in the asphalt mixture by X-ray CT (computed tomography) and 3D reconstruction.
3. Explore the effect of silicone on the moisture sensitivity and high-temperature stability of the asphalt mixture.
4. Determine the optimum silicone dosage for the OGFC mixture in consideration of permeability, distribution, performance of mixture, and economic cost.

## 2. Materials

### 2.1. Silicone Resin

**Figure 1:**
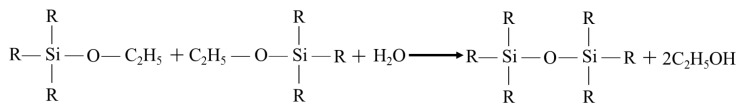
Reaction equation for preparing prepolymer of silicone resin.

**Figure 2:**
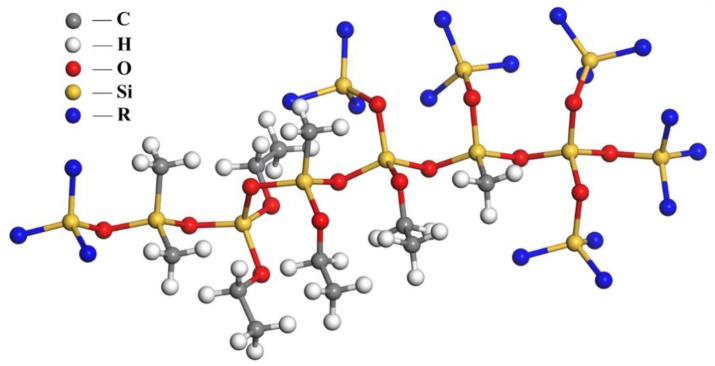
Structure of solidified silicone resin.

Silicone resin, in this study, was provided by cooperative enterprise Hubei Huanyu Chemical Co. Ltd. (Wuhan, China) Its preparation process is divided into two steps. Firstly, DMDES (dimethyldiethoxysilane), MTES (methyltriethoxysilane), TEOS (tetraethoxysilane), ethanol, and water were mixed at a certain ratio and polymerized for 5 h at 80 °C to obtain prepolymer of silicone resin. The reaction equation is shown in Figure 1, where R represents methyl, ethoxy, or the hydrogen atom. Secondly, two other silanes, IBTEO (isobutyltriethoxysilane) and APTES (aminopropyltriethoxysilane) were mixed with prepolymer as penetrating agent and tackifier, respectively. Liquid silicone resin product existing as low polymer can be obtained after completing the above steps. It will gradually solidify as ethanol solvent evaporates after it is sprayed on asphalt pavement, thereby exerting maintenance effect. The structure of solidified silicone resin is shown in Figure 2.

### 2.2. Asphalt, Aggregate, and Asphalt Mixture

Basalt from Jingshan, Hubei province (China), was used as an aggregate in this research. Basic physical properties are shown in Table 1. It can be found that all basic properties meet the criteria. SBS (styrene butadiene styrene) modified binder with penetration of 54 (0.1 mm at 25 °C, 100 g, and 5 s), ductility of 31.4 cm (5 cm/5 °C), and softening point of 78.6 °C was also used.

**Figure 3:**
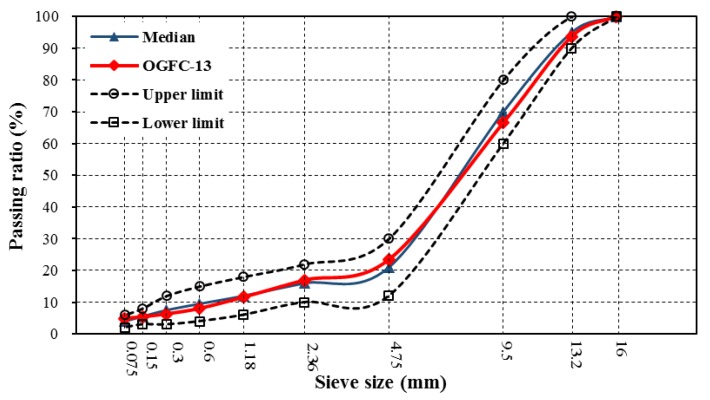
Gradation curve of open-graded friction course (OGFC)-13 asphalt mixture.

OGFC-13 (open-graded friction course) asphalt mixture was prepared to evaluate maintenance effect of silicone resin. It is a kind of hot-mix asphalt (HMA) overlay that has excellent drainage and noise reduction performance. The gradation curve is shown in Figure 3. The optimum asphalt content is 3.9% and air void is 19.2%.

## 3. Experimental Methods

### 3.1. Characteristics of Silicone Resin

The surface micromorphology, chemical composition and distribution of elements, and physical phase of solidified silicone resin were detected by QUANTA FEG 450 SEM (FEI, Hillsboro, OR, USA), Axios XRF (PANalytical B.V, Almelo, The Netherlands), JXA-8230 EPMA (JEOL, Akishima, Tokyo, Japan), and D8 Advance XRD (Bruker, Mannheim, Germany), respectively.

### 3.2. Permeability and Distribution of Silicone Resin in Mixture

The permeability and distribution of silicone resin in the mixture are key factors affecting maintenance effect. Permeability was measured by the time of silicone flowing through the mixtures, and distribution is marked by white paint in the resin after it is solidified as seen in a previous study [29]. This method can characterize the permeability and distribution to a certain extent, but lacks precision and intuitiveness. Furthermore, the distribution of silicone inside the samples cannot be detected. X-ray CT was applied in this study to accurately characterize the permeability and distribution of silicone resin in OGFC. X-ray CT is an important test method in medicine and industry. It irradiates the object via X-rays, and the tomographic image is obtained since every kind of material has its own radiodensity.

**Figure 4:**
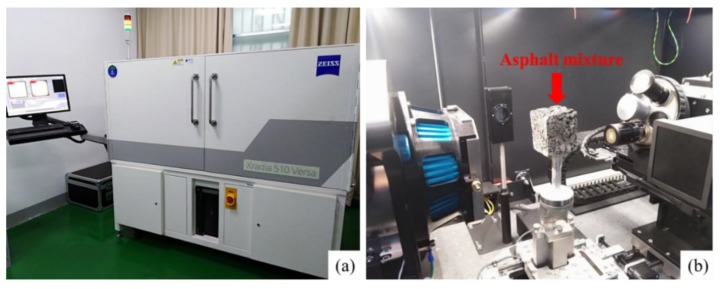
X-ray CT (**a**) and mixture been scanning (**b**).

Three kinds of dosages were set according to previous research: 200, 400, and 600 mL/m^2^. The mixture was scanned before spraying the silicone resin and after solidification. Samples are kept at 60 °C for 12 h after spraying for faster solidified speed. Furthermore, the mixture is cut into cubes with the size of 5 cm × 5 cm × 5 cm before CT scanning in order to obtain high image resolution, as shown in Figure 4. 3D reconstruction of pores and silicone resin was performed based on CT images to characterize the permeability and distribution of the silicone resin.

### 3.3. Moisture Susceptibility

**Figure 5:**
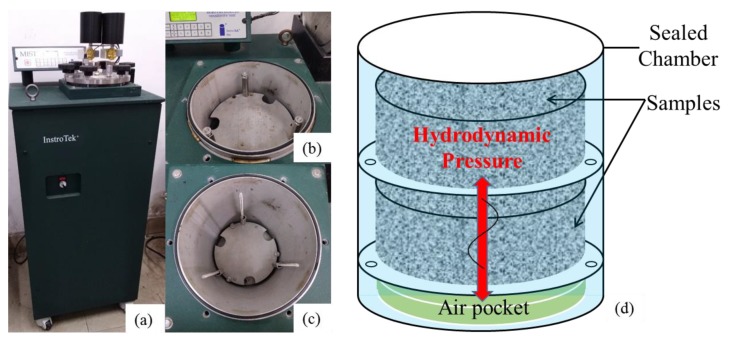
(**a**) Moisture induced stress tester (MIST); (**b**) top tray; (**c**) bottom tray; and (**d**) internal structure, with samples being treated.

MIST (moisture induced stress tester) was used to determine the effect of silicone resin on moisture susceptibility of the mixture. It can produce cyclical hydrodynamic pressure on the samples and simulate field moisture damage, as shown in Figure 5. Samples should be conducted by 3500 cycles of hydrodynamic pressure at 276 kPa and 60 °C, which meet ASTM D7870 (American Society of Testing Materials). In this study, the cycles were raised to 7000 to achieve a more severe degree of moisture damage, and thus more convincingly verifying the enhanced effect of silicone on moisture damage resistance of pavement.

The Marshall stabilities and indirect tensile strengths before and after MIST treatment were tested and recorded. The RMS (residual Marshall stability) and TSR (tensile strength ratio) were calculated to evaluate moisture susceptibility, as expressed by Equations (1) and (2):

(1) $$\mathrm{RMS}=MS_{2}/MS_{1}\times 100\%$$

(2) $$\mathrm{TSR}=ITS_{2}/ITS_{1}\times 100\%$$

where *MS*_1_ and *MS*_2_ are Marshall stabilities before and after moisture damage and *ITS*_1_ and *ITS*_2_ are indirect tensile strengths before and after moisture damage, respectively.

### 3.4. High-Temperature Performance

The effect of silicone resin on high-temperature performance was investigated by the rutting test and the Hamburg wheel tracking test (HWT), which were conducted in accordance to T0719-2011 in Chinese specification and AASHTO T324 (American Association of State Highway and Transportation Officials), respectively. HWT takes into account the effect of moisture on high-temperature performance compared with the rutting test. It can evaluate rutting resistance of the asphalt mixture under a moisture immersion condition, which is more similar to the field circumstance. The temperature, applied load, and loading rate were set at 60 °C, 0.7 MPa, and 42 cycles/min, respectively. Samples were all slabs with the size of 300 mm × 300 mm × 50 mm. The permanent deformations at 45 and 60 min were recorded as d_1_ and d_2_, respectively. The dynamic stability (DS) was calculated according to the following equation:

(3) $${\mathrm{DS}\;=\;(t}_{2}{\;-\;t}_{1}{)\;\times \;N/(d}_{2\;}{-\;d}_{1})$$

where *t*_1_ and *t*_2_ are 45 and 60 min, *N* is the loading rate of wheel.

## 4. Results and Discussion

### 4.1. Characteristics of Silicone Resin

#### 4.1.1. Surface Micromorphology

**Figure 6:**
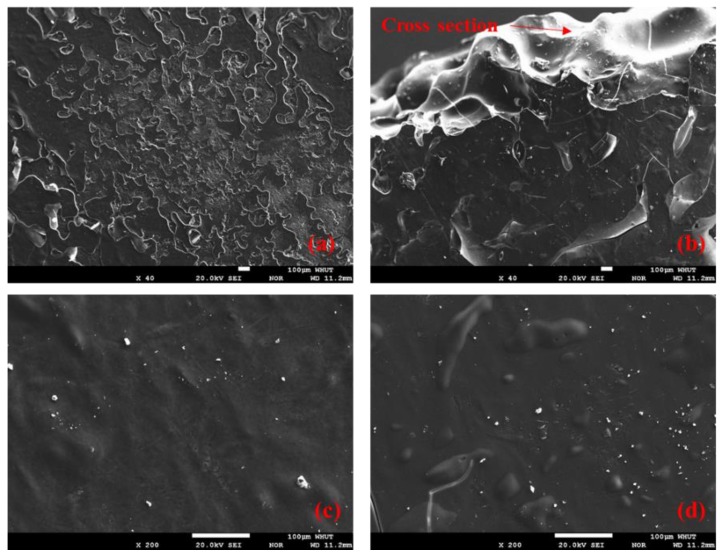
EPMA images of silicone resin: (**a**) surface of silicone at 40 magnification, (**b**) cross section at 40 magnification, (**c**,**d**) surface of silicone at 200 magnification.

**Figure 7:**
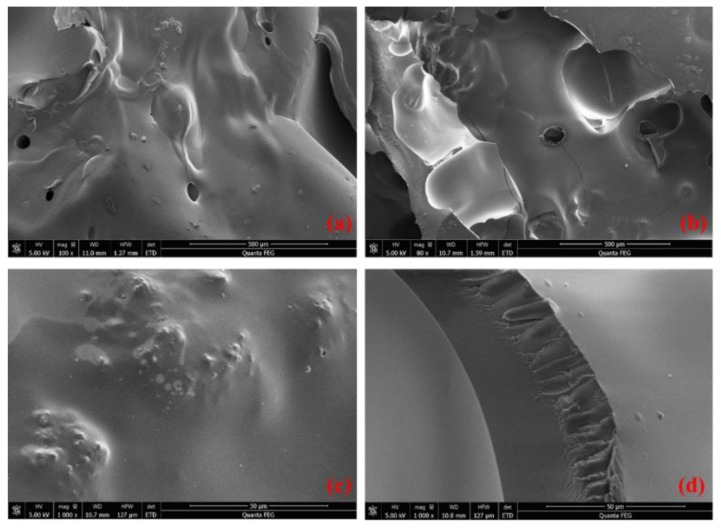
SEM images of silicone resin: (**a**,**b**) air pockets on silicone surface, (**c**) surface of silicone at 1000 magnification, (**d**) cross section at 1000 magnification.

The EPMA and SEM observations of silicone resin are presented in Figure 6 and Figure 7, respectively. It can be seen from Figure 6a that the surfaces of solidified silicone resin exhibit the appearance of rough scales, ensuring the skid resistance of pavement. The cross section shown in Figure 6b is smooth and without pores, which can effectively prevent moisture from permeating through the silicone resin film. It can be found that from Figure 6c,d that resin surface is flat and dense when the magnification is increased to 200. This surface characteristic can effectively isolate moisture, thereby improving moisture resistance of the asphalt mixture.

Some pores with size about 50 μm can be seen in Figure 7a,b. They are the air pockets produced by the evaporation of ethanol during solidification of silicone, which has a negative effect on the waterproof performance of silicone film. Reducing the evaporation rate of ethanol can suppress the formation of air pockets. Therefore, silicone resin fog seal maintenance should not be constructed at high temperatures to prevent silicone from solidifying too quickly and generating air pockets. Smooth and dense surface and a cross section of silicone can also be seen in Figure 7c,d at a magnification of 1000×, which makes the silicone have good moisture resistance.

#### 4.1.2. Element Composition and Distribution

XRF was used to detect the element composition of silicone resin. It is difficult to quantify elements with small atomic numbers due to the low X-ray yield of light elements, so the analysis is generally started from the Na. The results show that 99.5% of solidified silicone resin is Si except for the elements before Na.

**Figure 8:**
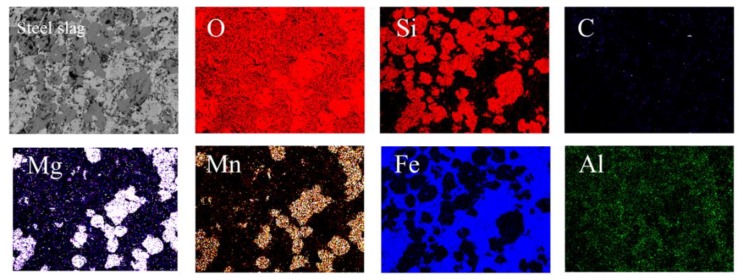
Elements maps of steel slag.

**Figure 9:**
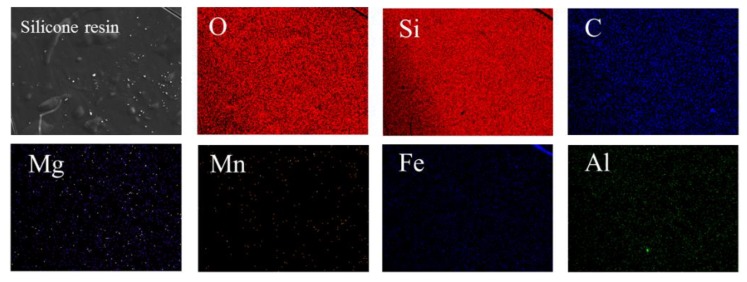
Elements maps of silicone resin.

EPMA was used to evaluate distribution of elements. In order to observe and analyze the distribution of elements in silicone more clearly, this study used solid waste steel slag with many types of elements as reference. The element maps of steel slag and silicone resin are presented in Figure 8 and Figure 9, respectively. The element distribution of steel slag was non-uniform except for oxygen, as shown in Figure 8. The reason is that steel slag contains various phases such as calcium forsterite, calcium silicate, and RO. On the contrary, it can be seen from Figure 9 that all elements are distributed quite evenly. It manifests that as the ethanol evaporates, the prepolymer in the liquid silicone is further polymerized to form a multidimensional Si–O–Si structure. In addition, the contents of elements other than C, O, and Si are very low, which is consistent with the results in the XRF.

#### 4.1.3. Physical Phase

**Figure 10:**
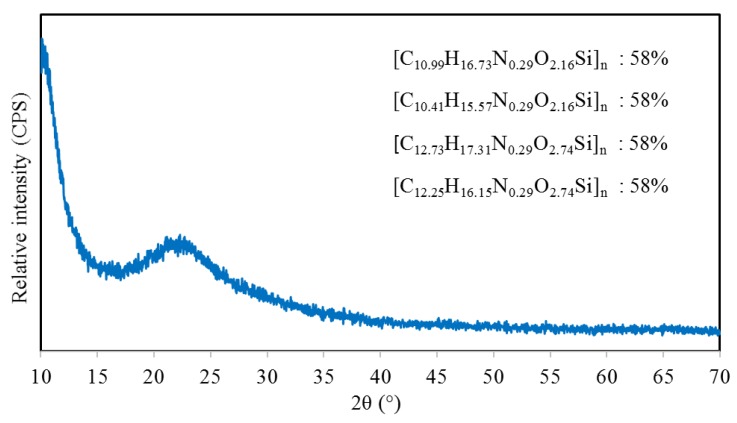
XRD results of silicone resin.

The solidified silicone resin was collected for XRD analysis. There are a variety of substances matching the XRD intensity peak due to the random combination of monomers during polymerization. Four substances that are most consistent with the XRD results are listed in Figure 10 with matching rates of 58%. XRD results imply that solidified silicone resin is the polymer consisting of C, H, N, O, and Si. The monomers of matching substances contain a small amount of N, which corresponds to the tackifier, APTES, used in the production of silicone resin. Furthermore, the number of atoms of C, H, N, O, and Si are approximately 10–13, 15–18, 0–1, 2–3, and 1 respectively, in the monomer of solidified silicone resin.

### 4.2. Permeability and Distribution of Silicone Resin

**Figure 11:**
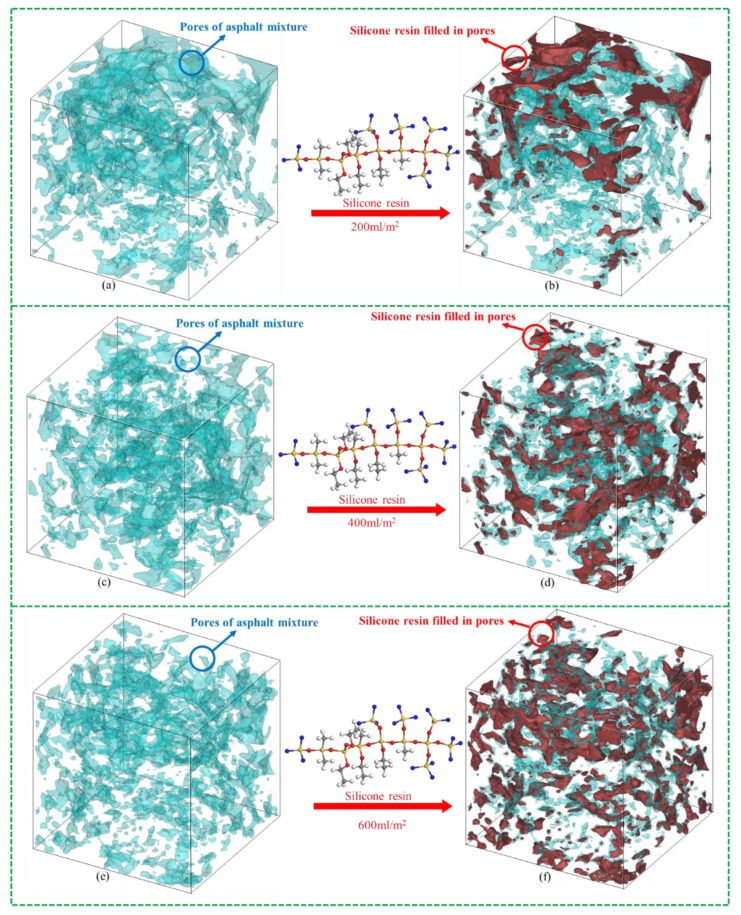
3D reconstruction of pores and silicone resin at dosages of 200 mL/m^2^: (**a**,**b**); 400 mL/m^2^: (**c**,**d**); and 600 mL/m^2^: (**e**,**f**).

Figure 11 presents 3D models of pores and silicone resin in OGFC mixture at dosages of 200, 400, and 600 mL/m^2^. Blue and red stand for pores and silicone resin, respectively. It could be found from Figure 11a,b that silicone resin only distributed on the top of the sample and did not penetrate into most of the pores at the dosage of 200 mL/m^2^. When the dosage was increased to 400 mL/m^2^, silicone resin was found to be substantially uniformly distributed throughout the pores of the sample, as shown in Figure 11c,d. Figure 11e,f show that the silicone resin is evenly distributed in the pores and more pores are filled by silicone when the dosage increases to 600 mL/m^2^.

The volumes of silicone and pores are calculated by 3D reconstruction software. The results show that the pore filling rates (percentages of silicone resin in all pores) were 23.5%, 39.8%, and 43.5% for dosages of 200, 400, and 600 mL/m^2^, respectively. It can be calculated that when the dosage was changed from 200 to 400 mL/m^2^, the pore filling rate increased by 16.3%, whereas the filling rate only increased by 3.7% when dosage increased from 400 to 600 mL/m^2^. The reason is that silicone resin only fills the pores on the surface due to the small dosage of 200 mL/m^2^. The increase of dosage to 400 mL/m^2^ significantly improved the pore filling effect, and more silicone is filled in the middle and bottom pores of the mixture. However, as the dosage of silicone increases to 600 mL/m^2^, excess liquid silicone flows away along the pores of the mixture before solidification since the air void OGFC is large. The increase of the pore filling rate is not remarkable. Therefore, 400 mL/m^2^ is the optimum silicone dosage for OGFC mixture in consideration of permeability, distribution, and economic cost.

### 4.3. Moisture Susceptibility

**Figure 12:**
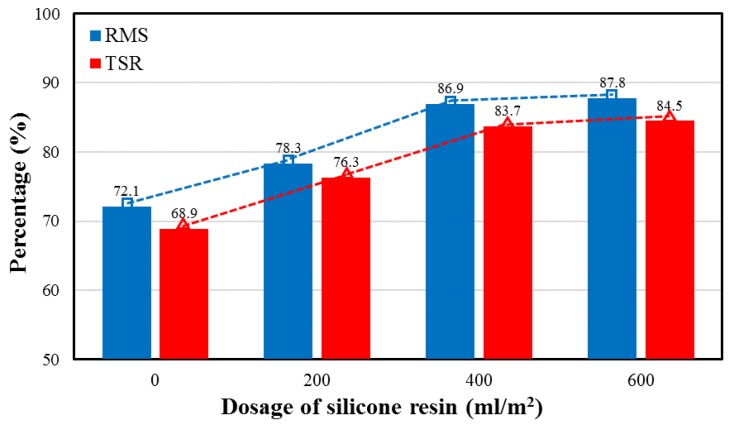
Moisture susceptibility of asphalt mixture at various silicone dosages.

Moisture susceptibility of the OGFC asphalt mixture at different silicone resin dosages is shown in Figure 12. It can be found that the moisture damage resistance of the asphalt mixture generally increases as the dosage of silicone increases. The moisture damage resistance of the mixture increases uniformly with the growth of dosage when the dosage was less than 400 mL/m^2^. However, as the dosage increased from 400 to 600 mL/m^2^, the growth rate in RMS and TSR slowed significantly, which is consistent with the distribution regularity of silicone in the mixture. The increase of dosage to 600 mL/m^2^ did not significantly improve the moisture damage resistance compared to 400 mL/m^2^ since the pore filling effect of silicone reached the upper limit. Therefore, 400 mL/m^2^ is the optimum silicone dosage for OGFC mixture in consideration of moisture susceptibility and economic cost.

### 4.4. High-Temperature Stability

**Figure 13:**
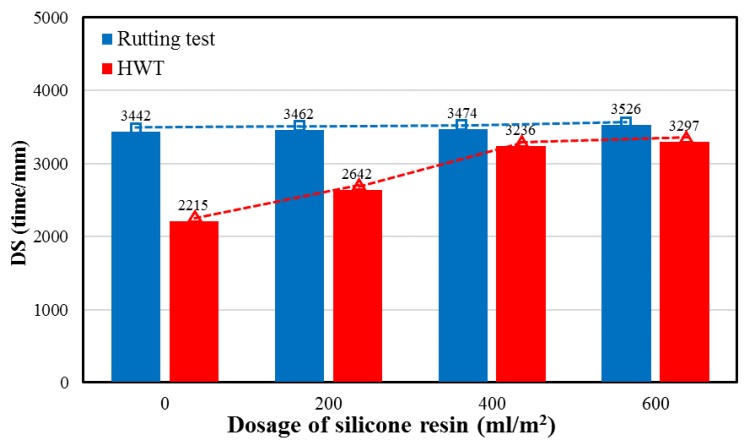
High-temperature stability of asphalt mixture at various silicone dosages.

Figure 13 presents high-temperature stability of the OGFC mixture at different silicone resin dosages. It can be clearly discerned that the dosage of silicone resin has little effect on the results of the rutting test, and the corresponding broken line (blue) is basically horizontal. This indicates that the silicone resin had no significant improvement on the rutting resistance of the asphalt mixture. Silicone resin solidified in the internal pores does not enhance the high-temperature stability of the mixture. A possible reason is that the silicone resin forms solidified film on the sample surface, which improves the rutting performance to some extent. However, the reduction of air voids could be a negative factor in terms of rutting. The combination of the two effects results in no obvious change in the rutting resistance. On the other hand, the results of HWT show a similar trend to moisture sensitivity. When the dosage was less than 400 mL/m^2^, the high-temperature stability of HWT increases with the growth of dosage. Nevertheless, the growth rate of high-temperature performance was significantly reduced when the dosage was more than 400 mL/m^2^. The reason is that the influence of moisture on high-temperature performance is taken into account in HWT, and high-temperature rutting resistance can be evaluated under moisture immersion condition. Therefore, the effect of silicone on the HTW results is significantly higher than the conventional rutting test due to the waterproof performance of silicone resin. It was also found that when dosages of silicone are the same, the dynamic stability of HWT is less than that of the conventional rutting test resulting from the influence of moisture.

## 5. Conclusions

In this study, physicochemical properties including surface micromorphology, element composition and distribution, and physical phase of solidified silicone resin were characterized. X-ray CT and 3D reconstruction technology were used to evaluate permeability and distribution of silicone resin in the asphalt mixture. Moisture sensitivity was detected by MIST. Rutting test and HWT were conducted to evaluate high-temperature performance. The following conclusions can be summarized:

1. The surface characteristic of silicone resin can effectively isolate moisture, thereby improving moisture resistance of the asphalt mixture. Elements are distributed quite evenly, indicating that the prepolymer in the liquid silicone is further polymerized to form a multidimensional Si–O–Si structure as the ethanol evaporates. Furthermore, the number of atoms of C, H, N, O, and Si are approximately 10–13, 15–18, 0–1, 2–3, and 1 respectively, in the monomers of solidified silicone resin.
2. Silicone resin only distributed on the top of the sample and did not penetrate into most of the pores at the dosage of 200 mL/m^2^. It was found to be evenly distributed throughout the pores of the sample when the dosage was increased to 400 or 600 mL/m^2^. The pore filling rate increased by 16.3% when the dosage was changed from 200 to 400 mL/m^2^, while it only increased by 3.7% when the dosage increased from 400 to 600 mL/m^2^. The reason is that excess liquid silicone flows away along the pores of the mixture before solidification.
3. Moisture damage resistance of the asphalt mixture generally increased as the dosage of silicone was increased. However, as the dosage was increased from 400 to 600 mL/m^2^, the growth rate in RMS and TSR slowed significantly since the pore filling effect of silicone reached the upper limit. The dosage of silicone resin had little effect on the results of the rutting test while it had a significant influence on HWT. The reason is that the moisture is taken into account in HWT. It was also found that when dosages of silicone are the same, the dynamic stability of HWT is less than that of conventional rutting test resulting from the influence of moisture.
4. In consideration of permeability, distribution, performance of mixture, and economic cost, it was found that 400 mL/m^2^ is the optimum silicone dosage for OGFC-13 mixture.

## Figures and Tables

**Table 1:** Basic physical properties of basalt.

Properties	Unit	Tested Value	Specification Used	Criteria in China
Specific gravity	g/cm^3^	2.855	ASTM C127	>2.6
Los Angeles abrasion loss	%	14.1	ASTM C535	<28
Water absorption ratio	%	0.67	ASTM C127	<2.0
Soundness	%	1.84	ASTM C88	<12
